# Identification of Biologically Effective Dose-Volumetric Parameters That Predict Radiation-Induced Hepatic Toxicity in Patients Treated With Helical Tomotherapy for Unresectable Locally Advanced Hepatocellular Carcinoma

**DOI:** 10.1097/MD.0000000000001904

**Published:** 2015-10-30

**Authors:** Jin Ho Song, Seok Hyun Son, Chul Seung Kay, Hong Seok Jang

**Affiliations:** From the Department of Radiation Oncology, Gyeongsang National University School of Medicine and Gyeongsang National University Hospital, Jinju (JHS); Department of Radiation Oncology, Incheon St. Mary's Hospital, College of Medicine, The Catholic University of Korea (SHS, CSK); and Department of Radiation Oncology, Seoul St. Mary's Hospital, College of Medicine, The Catholic University of Korea, Seoul, Korea (HSJ).

## Abstract

The purpose of this study is to identify dose-volumetric parameters that predict radiation-induced hepatic toxicity (RIHT) by analyzing the relationship between the biologically effective dose (BED) delivered to the normal liver and RIHT.

The clinical and dosimetric data from 123 patients with unresectable hepatocellular carcinoma (HCC) treated with helical tomotherapy were analyzed. The median radiation dose was a 50 Gy in 4.5 Gy fractions (range, 30–60 Gy in 1.8–5.0 Gy fractions) to 95% of the planning target volume. RIHT was defined as a Child-Pugh score increase of at least 2 points within 3 months of helical tomotherapy completion.

RIHT developed in 60 patients (48.7%). Multivariate logistic regression analysis showed that V_BED20_ (percentage of nontarget normal liver volume that received more than a BED of 20 Gy) was a significant parameter (*P* < 0.001), and the cut-off value was 40.8% with a sensitivity and specificity of 0.833 and 0.698, respectively, according to the receiver operating characteristic curve (*P* < 0.001).

Maintaining a V_BED20_ below 40.8% will reduce the risk of RIHT, and the proposed normal liver tolerance curve could be a useful guideline when treating unresectable HCC patients with various radiotherapy dose schedules.

## INTRODUCTION

Hepatocellular carcinoma (HCC) is the 5th most common cancer and the 3rd cause of cancer-related deaths worldwide after lung and stomach cancer.^1^ HCC is a heterogeneous disease, and thus, treatment strategies can vary according to the tumor stage as well as the patient's liver function.^2,3^ Although radical curative surgical resection is the treatment of choice in early-stage disease, many patients are inoperable or the tumor is unresectable at the time of diagnosis because of the tumor itself or poor liver function.^2^ In these patients, local therapies with transarterial chemoembolization (TACE), percutaneous ethanol injection (PEI), and radiofrequency ablation (RFA) have been used.^2,3^

However, radiotherapy (RT) has not been widely used due to the poor tolerance of the liver to radiation. In addition, a large additive margin to the gross tumor volume (GTV) was needed because of the technical difficulty in targeting a tumor that moves with respiration.^3,4^ This also makes it difficult to deliver higher radiation doses to the liver. Nowadays, both the advances in diagnostic imaging, including 4-dimensional computed tomography (4D-CT) and magnetic resonance imaging with gadolinium enhancement, and advances in radiation techniques such as intensity-modulated radiotherapy (IMRT), image-guided RT, stereotactic body radiotherapy (SBRT), or stereotactic radiosurgery (SRS) make it possible to irradiate tumors with higher doses. Recently, several studies have reported excellent results with various radiation techniques and doses.^5–12^

However, despite these studies, it remains difficult to predict radiation-induced hepatic toxicity (RIHT). Although several studies have reported a number of clinical and dosimetric predictive parameters for RIHT, it is difficult to draw a consistent conclusion due to the various definitions of RIHT, radiation techniques, and dose schedules.^4,13–19^

Identification of which definition of RIHT is the best and which radiation technique is most feasible is a complex problem that requires further investigations. However, different dose schedules can be relatively easily compensated. Theoretical estimates of the different radiation dose schedules as compared with normofractionation depend on the fractionation sensitivity of the normal liver, which can be quantified by the α/β ratio of the linear-quadratic model.^20^ In our previous study, we determined the α/β ratio of the normal liver of HCC patients was 8.^21^ Based on data from 98 patients who were treated with different fraction sizes (group A: 45–50 Gy in 4.5–5.0 Gy fractions and group B: 36–60 Gy in 2.5–3.0 Gy fractions), we concluded that the α/β ratio of the normal liver was 8.

Based on this earlier result, in this study, we further evaluated RIHT in advanced HCC patients who has been treated with helical tomotherapy and identified a parameter that could predict RIHT, using the biologically effective dose (BED) delivered to the normal liver.

## MATERIALS AND METHODS

### Patients

Between March 2006 and February 2012, 123 HCC patients, who were treated with helical tomotherapy at Seoul St. Mary's Hospital and Incheon St. Mary's Hospital, the Catholic University of Korea, were included in this study. All included patients satisfied the following criteria: unresectable locally advanced HCC; prior treatment with helical tomotherapy; age >18 years; pretreatment Child-Pugh (CP) class A or B; Eastern Cooperative Oncology Group (ECOG) performance status of 0, 1, or 2; 2 or more laboratory studies within 3 months after the completion of helical tomotherapy; 1 or more radiologic studies within 3 months after the completion of helical tomotherapy; and no intrahepatic disease progression within 3 months after the completion of helical tomotherapy. We retrospectively collected the patients’ clinical and dosimetric data following Institutional Review Board approval (IRB of Incheon St. Mary's Hospital, Catholic University of Korea; Reference number: OC13RISI0045).

The patients’ clinical characteristics are listed in Table 1. One hundred twenty-one patients (98.4%) had the evidence of any type of hepatitis, and 88 patients (71.5%) had liver cirrhosis. The pretreatment CP class was A in 96 patients (78%) and B in 27 patients (22%). The American Joint Committee on Cancer (AJCC) tumor stage (7th edition) was II in 13 patients (10.6%), III in 98 patients (79.7%), and IVA in 12 patients (9.8%). Prior to helical tomotherapy, 109 patients (88.6%) had undergone some type of local therapy, such as TACE, RFA, or PEI, and 15 patients (12.2%) had received systemic chemotherapy. TACE was performed in 108 patients (median, 2 times; range, 1–13 times), PEI in 10 patients (median, 2 times; range, 1–3 times), RFA in 9 patients (median, 1 time; range, 1–3 times), and systemic chemotherapy was administered to 15 patients. Within 3 months after the completion of helical tomotherapy, TACE was performed in 68 patients (55.2%; median, 2 times; range, 1–4 times). Additionally, RFA was performed in 2 patients (1.6%), PEI was performed in 3 patients (2.4%), and systemic chemotherapy was administered to 9 patients (7.3%).

**TABLE 1:**
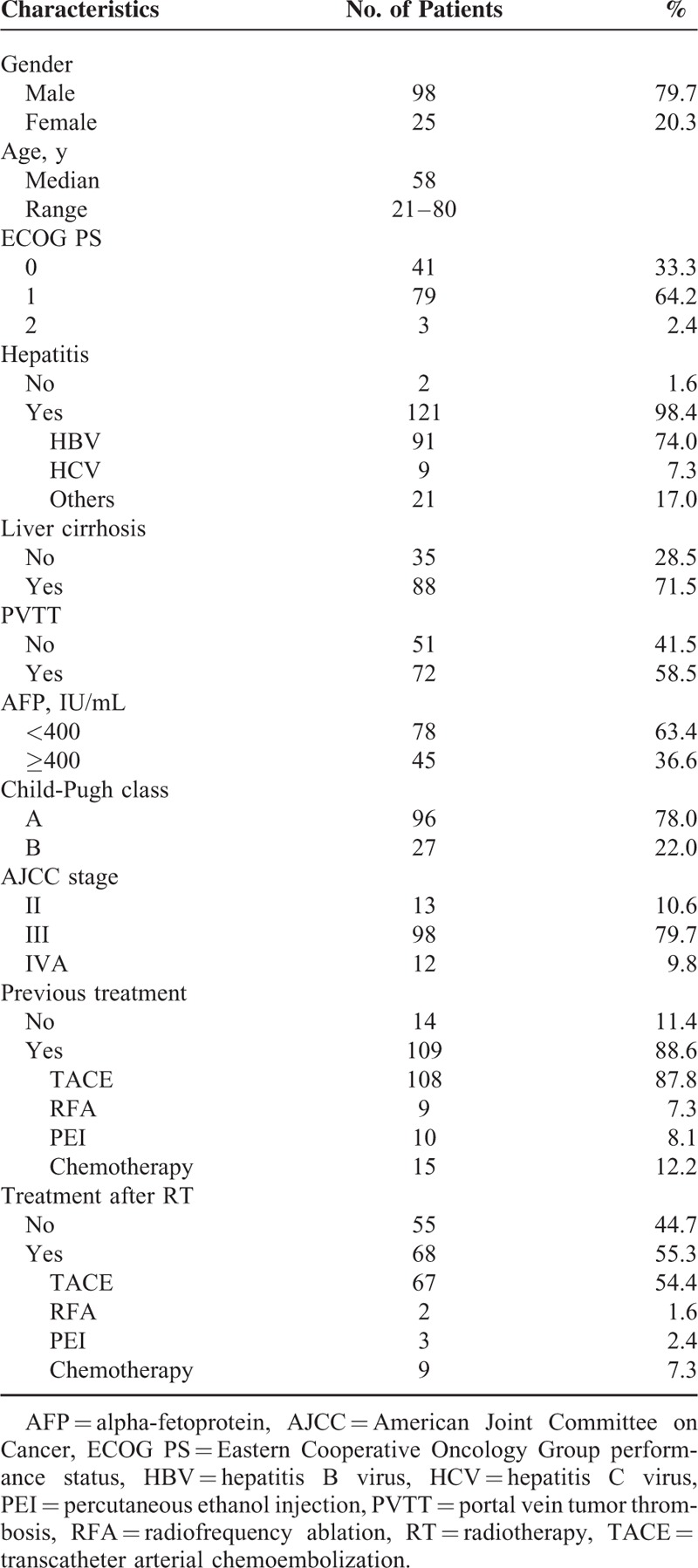
Patient Characteristics

### Radiation Treatment Techniques

For simulations, the patients were immobilized using the BodyFix system (Medical Intelligence GmbH, Schwabmunchen, Germany), in which the abdomen was compressed at a low pressure with foil. A spiral CT scan was then performed with intravenous contrast and a 2.5-mm slice thickness on a SOMATOM (Siemens, Berlin, Germany) or a LightSpeed RT16 (GE, Waukesha, WI) CT scanner.

The GTV was defined as a tumor that was enhanced in the arterial phase and diluted in the delayed phase. For the planning target volume (PTV), a 4D-CT scan was performed in 36 of 123 patients to generate an internal target volume in order to compensate for respiratory-induced liver movement after the installation of 4D-CT in March 2009 at Seoul St. Mary's Hospital and in March 2011 at Incheon St. Mary's Hospital. In the remaining 87 patients who did not undergo the 4D-CT scan, a 5 to 15 mm margin was added asymmetrically from the GTV to reduce the radiation doses to the stomach, duodenum, and small intestine. Several organs at risks were contoured, including the total liver, nontarget normal liver (NTNL), stomach, duodenum, intestine, both kidneys, and the spinal cord. The volume of NTNL was defined the volume of the total liver excluding the PTV.

The radiation dose was prescribed to 95% of the PTV with a median dose of 50 Gy, given in 4.5 Gy fractions (range, 36–60 Gy in 1.8–5.0 Gy fractions). Treatment planning was performed with the built-in software of the TomoTherapy Planning Station, which was used with TomoTherapy Hi-Art system (TomoTherapy, Madison, WI). A megavoltage cone-beam CT was performed before each daily treatment, and the inter-fractional patient setup error was corrected by a radiation oncologist.

### Definition and Evaluation of RIHT

RIHT was defined as an increase of at least 2 points in the CP score within 3 months after the completion of helical tomotherapy, as described in our previous studies.^19,21^ The CP class scoring system assesses liver dysfunction according to clinical and laboratory parameters. The parameters include serum albumin, billirubin levels, the prothrombin time, and the presence and degree of ascites or encephalopathy.

During radiation treatment, all patients were examined weekly by a physician. After treatment, all were followed up every 1 to 2 months and physical examinations and laboratory tests were performed at every visit in order to record the CP score variations.

### Parameters for Predicting RIHT

The clinical parameters included the age, gender, ECOG performance status, pretreatment CP class, AJCC stage, and pretreatment alpha-fetoprotein (AFP) level, as well as the presence of hepatitis, liver cirrhosis, portal vein tumor thrombosis (PVTT), and previous treatments.

The dosimetric parameters analyzed were the PTV, mean dose of NTNL, the percentage of NTNL volume receiving more than a BED of 5 Gy (V_BED5_), a BED of 10 Gy (V_BED10_), a BED of 15 Gy (V_BED15_), a BED of 20 Gy (V_BED20_), a BED of 25 Gy (V_BED25_), a BED of 30 Gy (V_BED30_), a BED of 35 Gy (V_BED35_), a BED of 40 Gy (V_BED40_), a BED of 45 Gy (V_BED45_), and a BED of 50 Gy (V_BED50_). The dose to the NTNL was adjusted for fraction size by converting each dose level to the BED. According to the linear-quadratic model, the BED was calculated with the following formula: 
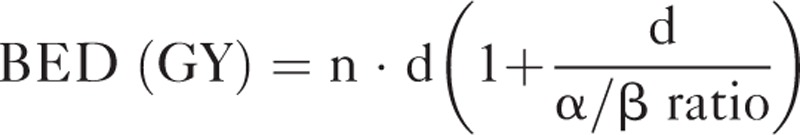


where n is the fractionation number, d is the daily dose, and the α/β ratio is assumed to be 8.

### Statistical Analysis

Pearson's Chi-squared test and the independent *t* test were used for univariate analysis to determine the clinical parameters associated with RIHT. A binary logistic regression was used for the univariate analysis of dosimetric parameters. For the multivariate analysis, the logistic regression model (stepwise forward) was used with all of the parameters that were significant in the univariate analysis. The receiver operating characteristic (ROC) curve was used to estimate significant dosimetric parameters and to obtain the most meaningful cut-off value. A *P* value of <0.05 was considered significant.

## RESULTS

### Parameters for Predicting RIHT

RIHT was observed in 60 of 123 patients (48.8%) within 3 months after helical tomotherapy completion. The results of the univariate analysis of clinical parameters are shown in Table 2. Of the several clinical parameters, AJCC tumor stage and the presence of PVTT were significant parameters for predicting RIHT (*P* = 0.005 and 0.012, respectively). Other parameters including age, gender, ECOG performance status, pretreatment CP class, pretreatment AFP level, presence of hepatitis or liver cirrhosis, previous treatments, and treatment after RT did not influence the possibility of RIHT.

**TABLE 2:**
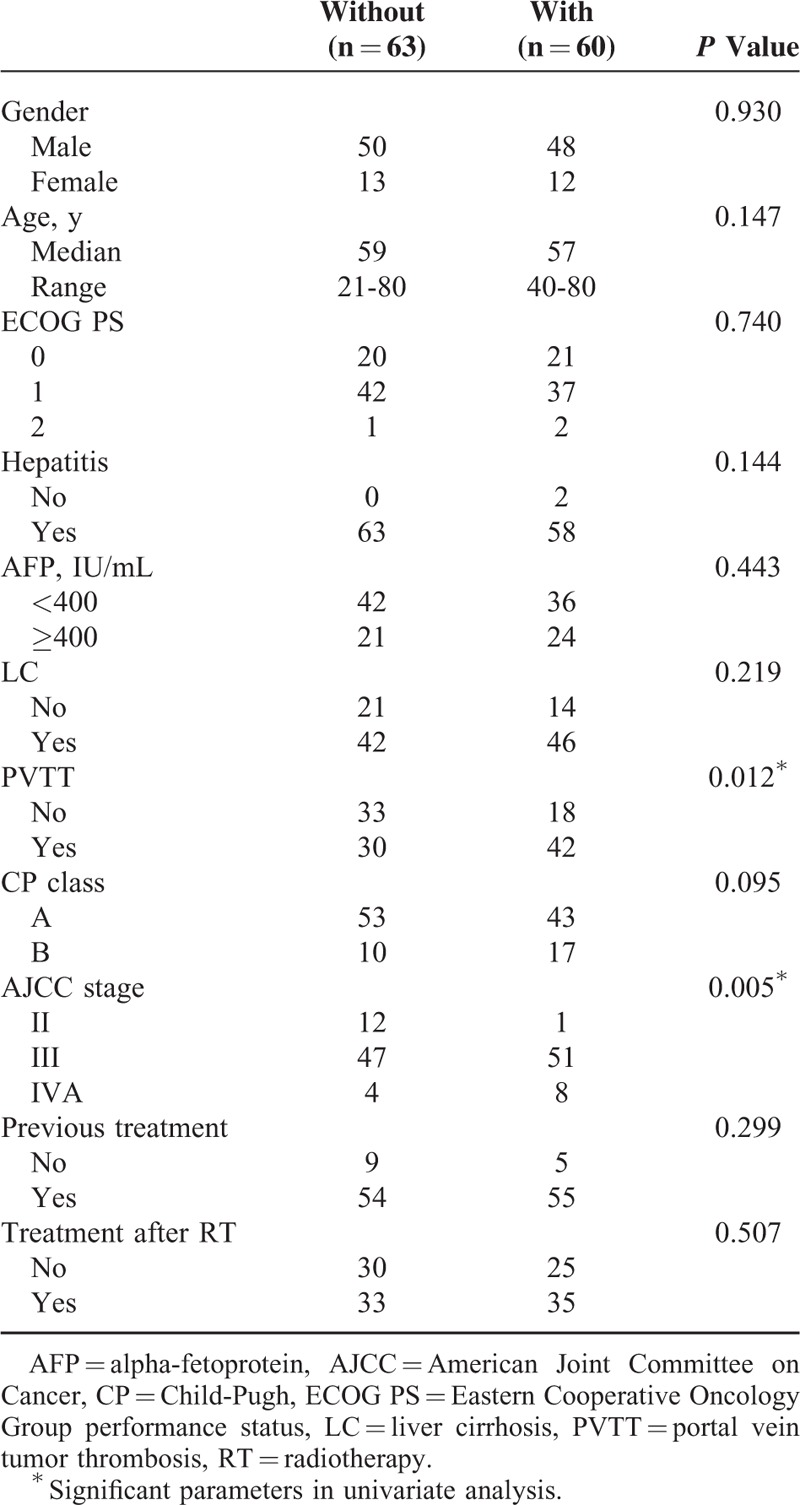
Clinical Parameters in Patients With or Without an Increase in Child-Pugh Score ≥2

The univariate analysis of dosimetric parameters showed that all parameters except for PTV were significant. The mean dose of NTNL, V_BED5_, V_BED10_, V_BED15_, V_BED20_, V_BED25_, V_BED30_, V_BED35_, V_BED40_, V_BED45_, and V_BED50_ were all associated with RIHT (Table 3). The mean dose of NTNL was significantly higher in the patients with RIHT than in those without RIHT (20.1 ± 3.3 Gy vs 15.4 ± 4.2 Gy, *P* < 0.001).

**TABLE 3:**
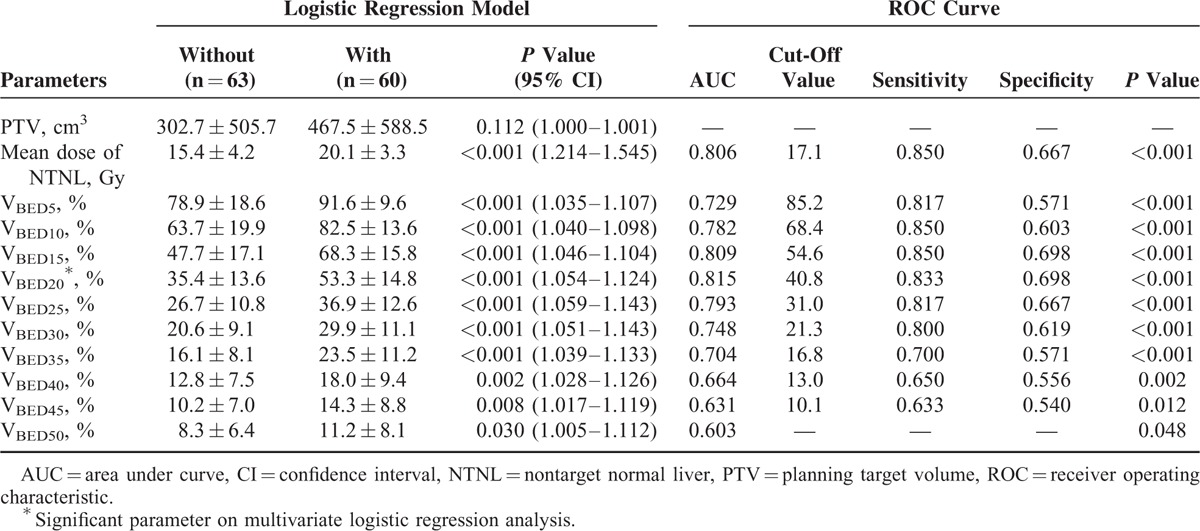
Dosimetric Parameters in Patients With or Without an Increase in Child-Pugh Score ≥2

**FIGURE 1:**
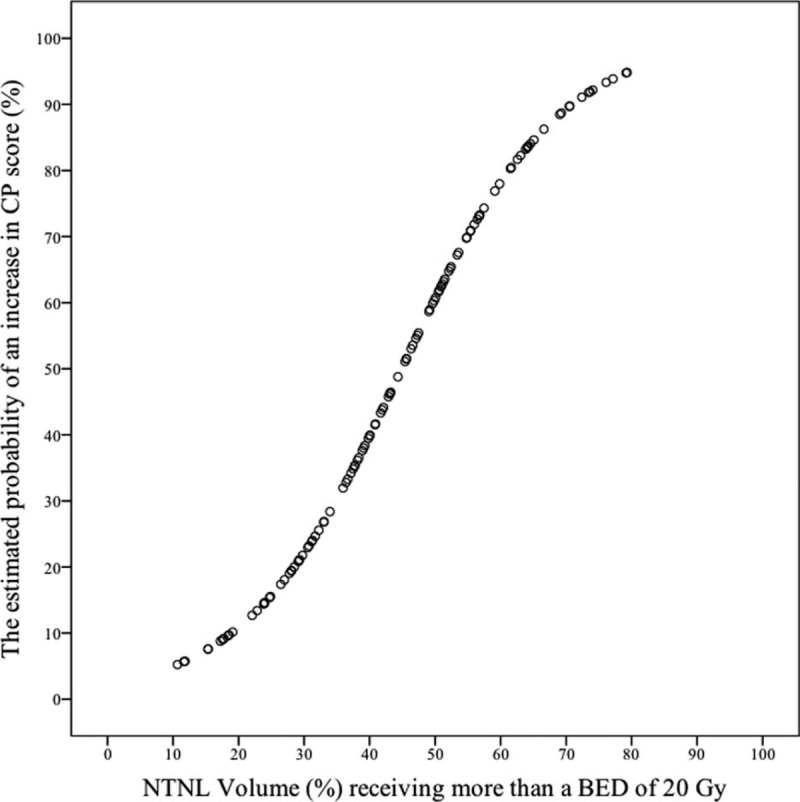
Estimated probability curve of radiation-induced hepatic toxicity for V_BED20_. The probability of radiation-induced hepatic toxicity increase as a sigmoid shape according to the increase of the nontarget normal liver volume receiving more than a biologically effective dose of 20 Gy.

**FIGURE 2:**
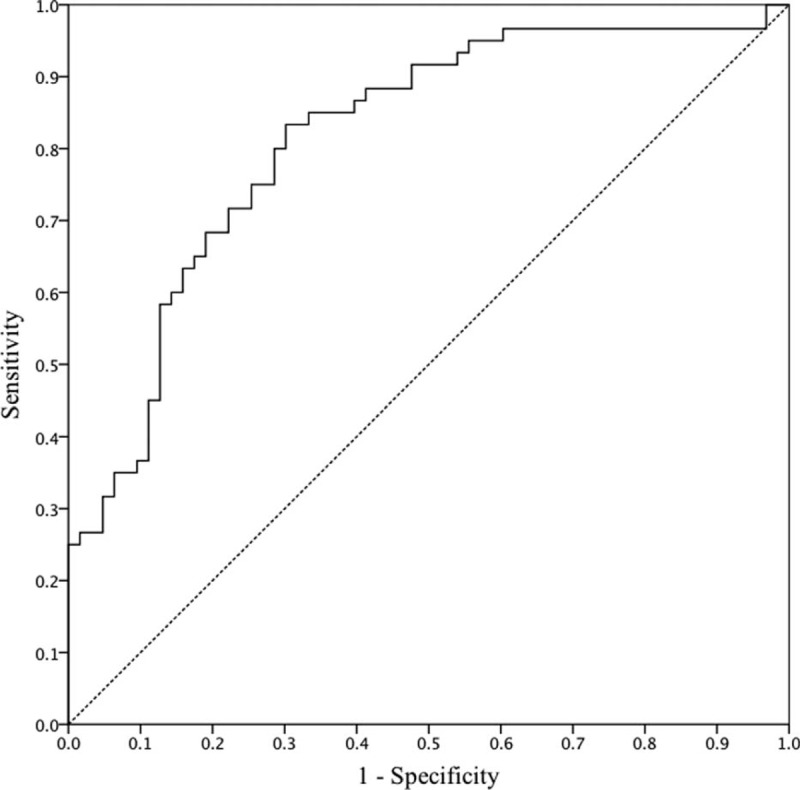
Receiver operating characteristic (ROC) curve for V_BED20_. The area under the ROC curve was 0.815, and the optimal cut-off value for V_BED20_ was 40.8%, with a sensitivity of 0.833 and a specificity of 0.698.

In the multivariate analysis, V_BED20_ was the only significant parameter predictive of RIHT (*P* < 0.001). The estimated probability curve and ROC curve of V_BED20_ is shown in Figures 1 and 2, respectively. The area under the ROC curve (AUC) was 0.815 for V_BED20_ (*P* < 0.001), and the most optimal cut-off value of V_BED20_ was 40.8%, with a sensitivity of 0.833 and a specificity of 0.698 (Table 3). RIHT was observed in 10 of 53 patients (18.8%) with a V_BED20_ of ≤40.8%, and in 50 of 70 patients (71.4%) with a V_BED20_ of >40.8%. For V_BED20_ with a cut-off value of 40.8%, the accuracy was 0.756 (93/123).

### Dose-Volume Histogram of Normal Liver That Predicts RHIT

Understandably, close correlations were found between the dosimetric parameters. The correlation coefficient values between the dosimetric parameters were in a range of between 0.915 and 0.990 (V_BED5_ vs V_BED10_, *r* = 0.915, *P* < 0.001; V_BED10_ vs V_BED15_, *r* = 0.929, *P* < 0.001; V_BED15_ vs V_BED20_, *r* = 0.956, *P* < 0.001; V_BED20_ vs V_BED25_, *r* = 0.949, *P* < 0.001; V_BED25_ vs V_BED30_, *r* = 0.951, *P* < 0.001; V_BED30_ vs V_BED35_, *r* = 0.935, *P* < 0.001; V_BED35_ vs V_BED40_, *r* = 0.934, *P* < 0.001; V_BED40_ vs V_BED45_, *r* = 0.987, *P* < 0.001; V_BED45_ vs V_BED50_, *r* = 0.990, *P* < 0.001). Although V_BED20_ was the only significant parameter in the multivariate analysis, we further tested the predictability of each parameter with the ROC curve because other parameters were also significant in the univariate analysis, and the AUC showed meaningful and statistically significant results. These results are shown in Table 3.

**FIGURE 3:**
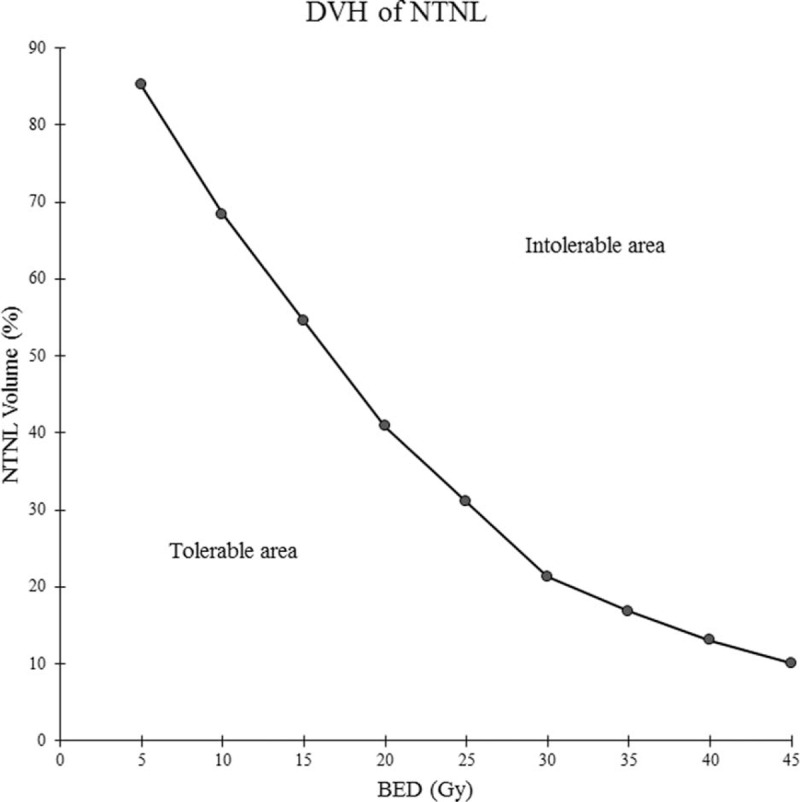
Tolerance curve, expressed as a biologically effective dose-volume histogram for hepatocellular carcinoma. The dose-volume histogram is suggested to be in the tolerable to reduce radiation-induced hepatic toxicity.

The AUC of each parameter was highest for the V_BED20_ (0.815, *P* < 0.001), and lowest for the V_BED50_ (0.603, *P* < 0.048). We excluded V_BED50_ when determining the cut-off value because of the low AUC value and the relatively low statistical significance, compared to the other parameters. We selected the optimal cut-off value of each parameter to draw the tolerance curve. These value was 85.2% for V_BED5_, 68.4% for V_BED10_, 54.6% for V_BED15_, 40.8% for V_BED20_, 31.0% for V_BED25_, 21.3% for V_BED30_, 16.8% for V_BED35_, 13.0% for V_BED40_, and 10.1% for V_BED45_. By connecting these cut-off values, we obtained the tolerance curve that can predict the possibility of RIHT in HCC patients who were treated with helical tomotherapy (Fig. 3).

## DISCUSSION

RIHT is one of the most important dose-limiting complications when treating HCC patients with RT. Since the liver is an important organ with numerous functions, a severe reduction in liver function can delay consequent treatment and can also directly affect survival.^3,4,22^

Several recent studies have reported risk factors of RIHT, although the definition of RIHT varied. Dawson and Ten Haken^14^ analyzed 203 patients with primary and metastatic liver tumors using 3-dimensional conformal radiotherapy (3D-CRT). The authors considered RIHT as a Radiation Therapy Oncology Group grade 3 or worse hepatic toxicity, and found that a mean dose with a cut-off value of 31 Gy was significant and also that treated patients with metastatic tumors were more vulnerable to RIHT than were primary liver cancer patients. Cheng et al^23^ analyzed 89 HCC patients who were treated with 3D-CRT. The mean tumor dose was 49.9 Gy (range, 36–66 Gy) in daily fractions of 1.8 to 3.0 Gy. The authors considered RIHT as Grade 3 or worse hepatic enzyme elevation according to the Common Terminology Criteria for Adverse Events (CTCAE), and found that the mean liver dose was significantly greater in patients with RIHT (22.9 vs 19.0 Gy, *P* = 0.05) and also that hepatitis B virus status and CP class B were clinical risk factors for RIHT development. Kim et al^15^ treated 105 HCC patients with 3D-CRT. The median tumor dose was 54 Gy (range, 44–54 Gy) with a fraction size of 2 to 3 Gy. They also used CTCAE criteria for RIHT. The authors found that the total liver volume receiving more than 30 Gy was the only significant parameter of RIHT prediction, and suggested that the volume should be limited to ≤60%. Liang et al treated 109 primary liver tumor patients with hypofractionated 3D-CRT.^17^ The median dose was 54 Gy (range, 38–68 Gy) with a median fraction size of 4.6 Gy (range, 4–6 Gy). The authors used the CTCAE criteria when defining RIHT. The liver volume receiving ≥20 Gy was the most significant dosimetric parameter, with a cut-off value of 48.5%. In our previous study based on data from the 72 HCC patients who were treated with helical tomotherapy (40–50 Gy in 4–5 Gy fractions), we concluded that the normal liver volume receiving ≥15 Gy was the most significant factor when predicting a CP score increase of 2 points.^19^

Despite these recent studies, it remains difficult to obtain a consistent and conclusive identification of the dosimetric parameters that are predictive of RIHT because of the various radiation doses, definitions of RIHT, and radiation techniques. To overcome this limitation, we performed the present study. Our previous study defined the α/β ratio of normal liver as 8 in HCC patients.^21^ Some researchers used 2 or 2.5 as the α/β ratio of normal liver, and some used 10 to calculate the BED delivered to the normal liver^13,15,23,24^; this inconsistency seems to derive from a lack of studies that define the α/β ratio of normal liver. Based on our previous result, the dose to the NTNL was converted to the BED, and thus, our dosimetric parameter was presented in BED scale. Kim et al^15^ demonstrated that V_30_ was a significant parameter in patients who were treated with conventional fractionated RT, and Liang et al^17^ demonstrated that V_20_ was a significant parameter in patients who were treated with hypofractionated RT (4–6 Gy per fraction). In our study, V_BED20_ was a significant parameter and its cut-off value was 40.8%, and these were lower than those reported in the above-mentioned studies (60% for V_30_ in the study by Kim et al and 48.5% for V_20_ in the study by Liang et al).

Some caution is needed in order to interpret our results, although they can be used widely. First, we defined the RIHT as an increase of 2 points in the CP score. Liaw et al^25^ also used an increase in the CP score to evaluate the deterioration of hepatic function in patients treated with lamivudine. The Quantitative Analyses of Normal Tissue Effects in the Clinic (QUANTEC) report also described an increase in the CP score of 2 or more points as an indicator of RIHT and suggested that the pretreatment and posttreatment CP scores should be recorded.^18^ Our previous studies also showed that the CP score progression was a useful indicator of RIHT and an important dose-limiting factor in patients treated with RT.^19,26,27^ Second, only patients who were treated with helical tomotherapy were included in this study. The planning and delivery method of helical tomotherapy is quite different from that of 3D-CRT or static IMRT. Since helical tomotherapy delivers a continuous beam from all angles by rotating a ring gantry,^28^ a low to moderate dose of radiation is delivered to a much wider region of the liver. As shown above, in this study, the significant parameter and its cut-off value are lower than those of other studies. This is likely due to the characteristics of helical tomotherapy. Therefore, caution is needed when directly applying our results to 3D-CRT or static IMRT cases. Third, with the recent advancements in radiation techniques, hypofractionated RT has also been widely used in HCC treatment. A fraction size of 4 to 6 Gy is commonly used with good results.^5,8,12,26,29^ Our study can be a guideline for such clinical practice to predict RIHT with any dose schedules. However, when a fraction size more than 8 to 10 Gy is used for SBRT or SRS, caution seems necessary because the radiobiology of SBRT or SRS are thought to be different and does not follow the linear-quadratic model.^30,31^

## CONCLUSION

In conclusion, RIHT can be predicted with the normal liver volume receiving a dose more than a BED of 20 Gy (V_BED20_) with the α/β ratio of 8. Maintaining a V_BED20_ below 40.8% will reduce the risk of RIHT, and the proposed normal liver tolerance curve could be a useful guideline when treating HCC patients with various RT dose schedules.
